# The Inter-Alveolar Pores of the Lung

**Published:** 1907-06-29

**Authors:** 


### Points in Pathology.

### THE INTER-ALVEOLAR PORES OF THE LUNG.

### Their Role in Emphysema and in the Spread of Pneumonia.

It is a well-known observation in microscopical sections of pneumonic lung that strands of fibrin often appear to extend from one alveolus to the next through minute holes in the inter-alveolar walls. It has been argued by some that these minute holes, or inter-alveolar pores, do not exist in healthy lungs; and that in pneumonia they are produced by the inflammatory change itself causing minute ulcers which here and there perforate through so as to put one alveolus in direct communication with the next.

Upon this view it has always been difficult to understand how the pneumonic process is able to spread so quickly through a whole lobe of a lung, especially when the microscope shows little or no simultaneous inflammation of the bronchioles or of the inter-alveolar connective tissue. It is remarkable how extensive the inflammatory trouble is within the alveoli, and how little of it there is in the other structures of the lung. If one alveolus does not normally communicate with the next, it is difficult to understand how the spread takes place from alveolus to alveolus. Conceivably the trouble might spread from one group of alveoli up into a bronchiole, and thence down into the next group of alveoli, and so on; but in this case the bronchioles should show some evidence of inflammation, which they seldom do. We are speaking of lobar pneumonia, and not of broncho-pneumonia, of course.

#### Hansemann's Experiments.

Experimental investigation upon the existence of inter-alveolar pores in healthy lungs is by no means easy, but Hansemann, as long ago as 1895, succeeded in demonstrating them well. His method depended upon using a simple intratracheal injection, sections of the lung being subsequently prepared and examined microscopically. There are two excellent pictures of such sections in the "Mittheilungen der Akademie der Wissenschaften zu Berlin," vol. ix., 1895, page 453, showing definite but minute pores through which each alveolus communicates with the next. These are no doubt the same pores through which fibrin strands are seen passing in pneumonic lung, and their existence makes it easy to understand how the pneumococcal process which begins in one alveolus may spread rapidly from it to the surrounding alveoli, and so, by direct extension, all over the lobe of a lung.

#### Hypertrophic Emphysema.

The presence of inter-alveolar pores in normal lung also makes it easier to understand the changes that occur in hypertrophic emphysema. In this condition, as is well known, there is not only defective elasticity of the connective tissue of the lung, and over-distension of all the alveoli, but the latter are also diminished in number and enormously increased in size by several adjacent alveoli being thrown into free communication with one another. This intercommunication is usually described as due to breaking down of inter-alveolar walls. It has been difficult to explain why the latter should thus break asunder in emphysema, as they undoubtedly do; but if the existence of inter-alveolar pores in normal lungs be allowed, the explanation is not so far to seek. When, from the adoption of a more and more inspiratory position of the chest the lungs become more and more expanded, the minute pores will be stretched until they become holes between the alveoli; and if the stretching goes on the margins of these holes will tear until all the appearances seen in a microscopical section of emphysematous lung will be produced.

